# Porous aromatic framework electrodes boost electrochemical uranium extraction

**DOI:** 10.1021/acscentsci.3c01635

**Published:** 2024-01-09

**Authors:** Shuo Zhang, Hui Li, Shuao Wang

**Affiliations:** State Key Laboratory of Radiation Medicine and Protection, School for Radiological and Interdisciplinary Sciences, and Collaborative Innovation Center of Radiation Medicine of Jiangsu Higher Education Institutions, Soochow University, Suzhou 215123, China

Uranium extraction from seawater (UES) is crucial for the sustainable development of nuclear energy. Electrochemical methods, especially the electrocatalytic approach, represent promising technologies capable of large-scale UES without thermodynamic-equilibrium limitation. However, conventional electrode materials have impeded the electrochemical strategies in UES due to their lack of stability, efficiency, or scalability. Now, Zhu, Zhao, and their collaborators have reported groundbreaking work detailing self-standing flexible porous aromatic framework (PAF)-based electrodes which may address these issues. This innovative material designed at the molecular level significantly expands the feasibility of electrochemical UES.^1^

Uranium, a redox-active metal, exhibits the capability of undergoing reduction from the highly soluble uranyl(VI) ion to the almost insoluble U(IV).^2^ This phenomenon provides an alternative pathway for potential breakthroughs in the field of UES. Research conducted by Cui’s group first demonstrated that the half-wave rectified alternating current electrochemical (HW-ACE) method can disrupt the complex equilibrium by effectively converting U(VI) to U(IV) through the utilization of amidoxime-functionalized carbon electrodes.^3^ Subsequently, Wang and co-workers demonstrated the efficacy of electrocatalysis using an amidoxime-functionalized nanoscale catalyst metal–nitrogen–carbon (M–Nx–C–R) composition.^4^ Despite this progress, a significant gap persists in the field of UES. This gap can be attributed to two factors. First, active compounds commonly used in UES electrodes, typically micro/nanomaterials, tend to detach from binders after undergoing multiple processing steps. Second, the lack of molecular-level design in composite electrode materials impedes the full development of functional groups and conductive components within the materials. Consequently, there is an urgent demand for meticulous design and the synthesis of stable and easily scalable integrated electrode materials to drive forward the implementation of electrocatalytic UES technology.

In Zhu’s latest research published in *ACS Central Science*,^1^ flexible self-standing, binder-free, metal-free electrodes were fabricated (PAF-144-PE) by a scalable electropolymerization method. Based on the rational design, adsorption sites, catalytic sites, abundant mass transfer channels, and electric field assistance are integrated into the electrodes. Uranyl ions are selectively captured and subsequently transformed into Na_2_O(UO_3_·H_2_O)_*x*_ in the presence of Na^+^ via reversible electron transfer, achieving a removal efficiency exceeding 98.0%. The synergistic effect of adsorption and catalysis at the molecular level enhances their combined impact, thereby improving overall UES performance. The electrochemical process exhibits kinetics 3 times faster than the physicochemical adsorption process, demonstrating an exceptionally high removal capacity at low concentrations (1413.9 mg g^–1^ at 4.6 mg L^–1^), surpassing most reported materials for uranium extraction. In the natural seawater test, a high uranium extraction capacity of 12.6 mg g^–1^ was achieved after 24 days of operation (Figure 1). Control experiments emphasize the indispensable role of synergistic effects between adsorption and catalytic sites in constructing highly efficient electrodes for UES, as the removal capacity of the dual-function electrode PAF-E surpasses that of the corresponding single-function electrode (adsorption or catalysis function only) by 2.34-fold or 1.40-fold, respectively. The outstanding outcomes establish a fresh standard for electrochemical UES and pave the way for the advancement of comprehensive electrode materials for large-scale implementation.

**Figure 1:**
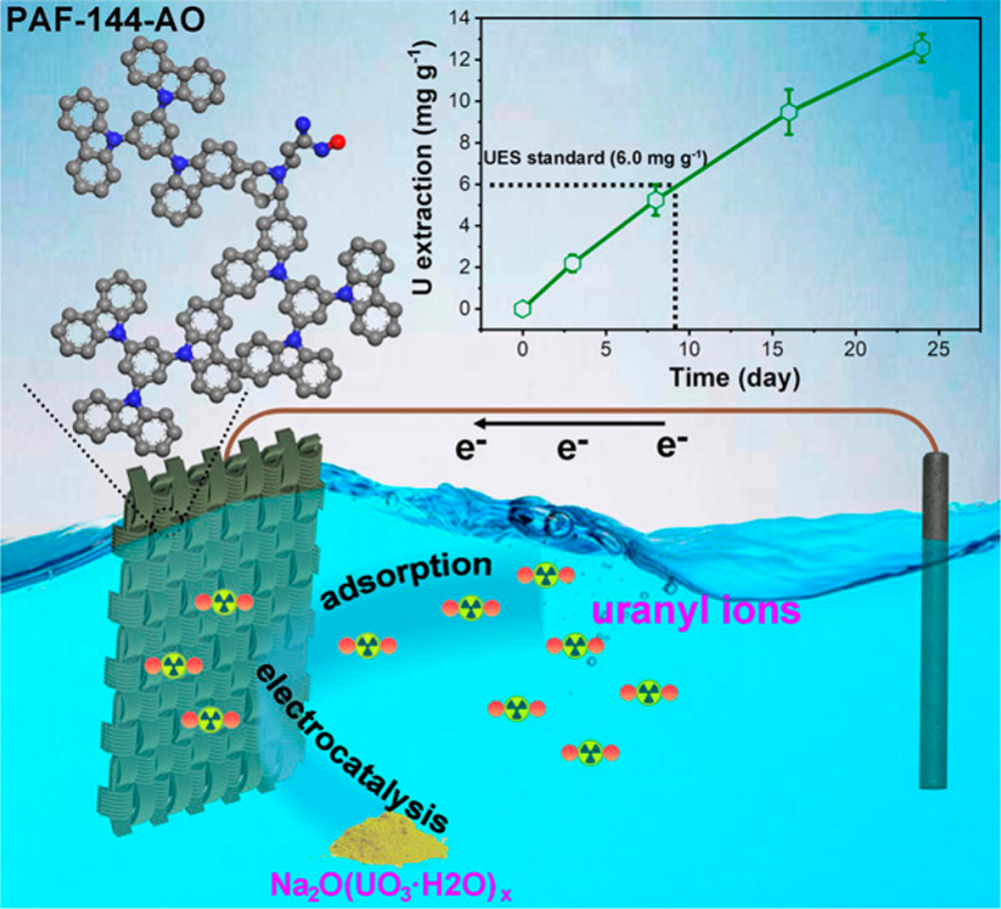
Schematic of self-standing porous aromatic framework electrodes (PAF-E) extracting uranium from seawater. The inset shows the uranium extraction capability of PAF-E from natural seawater. Reproduced with permission from ref (1). Copyright 2023 American Chemical Society.

The study offers a promising and comprehensive solution for UES. The remarkable performance in uranium extraction was achieved through the collaborative improvement of amidoxime groups, electroactive sites, and porous frameworks, employing a synergistic approach that combines adsorption and electrocatalysis. The development of comprehensive electrode materials, capable of large-scale production, has significantly matured electrocatalytic seawater technology and expands the technical reserves within the realm of UES.

Significant progress has been achieved in enhancing the overall performance of materials utilized in electrochemical uranium extraction, owing to persistent efforts and extensive research. Notably high uranium extraction capacities have been attained under laboratory conditions. Nevertheless, the practical implementation of electrochemical UES continues to face multiple challenges. Until now, electrochemical uranium extraction studies have commonly employed excessive bias voltage (−5 or −3 V), complicating our understanding of the uranium deposition mechanism. This complexity arises due to the potential increase in local alkalinity around the cathode induced by water electrolysis, leading to uranium hydrolysis precipitation products.^5^ To enhance the comprehension of uranium deposition mechanisms and reduce energy consumption and operational costs, future research in electrochemical uranium extraction studies should focus on designing and fabricating electrode materials that enable low bias voltage operation. Furthermore, electrocatalytic extraction methods often rely on preadsorption for uranium. However, in natural seawater, the extremely low concentration (∼3.3 ppb) and harsh chemical environment render functional groups ineffective.^6,7^ Exploring strategies to overcome the thermodynamic constraints imposed on U(VI) pre-enrichment requires careful consideration.^8,9^ Studies on uranium enrichment through catalytic reduction strategies frequently overlook the primary form of uranium species present in seawater, UO_2_(CO_3_)_3_^4–^, which exhibits significantly distinct physical and chemical properties compared to those of UO_2_^2+^.^10^ Therefore, the design and preparation of porous materials with scalability, superb electrical conductivity, an abundance of selective functional groups, and excellent electrocatalytic reduction for UO_2_(CO_3_)_3_^4–^ can have an exciting impact on UES and contribute to the sustainable development of nuclear energy.

## References

[ref1] Chen D.; Li Y.; Zhao X.; Shi M.; Shi X.; Zhao R.; Zhu G. Self-Standing Porous Aromatic Framework Electrodes for Efficient Electrochemical Uranium Extraction. ACS Cent. Sci. 2023, 9, 2326 10.1021/acscentsci.3c01291. 38161362 PMC10755849

[ref2] Li H.; Wang S. Reaction: Semiconducting MOFs Offer New Strategy for Uranium Extraction from Seawater. Chem. 2021, 7 (2), 279–280. 10.1016/j.chempr.2021.01.013.

[ref3] Liu C.; Hsu P.; Xie J.; Zhao J.; Wu T.; Wang H.; Liu W.; Zhang J.; Chu S.; Cui Y.; Half-wave A. Rectified Alternating Current Electrochemical Method for Uranium Extraction from Seawater. Nat. Energy. 2017, 2 (4), 17007–17015. 10.1038/nenergy.2017.7.

[ref4] Yang H.; Liu X.; Hao M.; Xie Y.; Wang X.; Tian H.; Waterhouse G. I. N.; Kruger P. E.; Telfer S. G.; Ma S. Functionalized Iron-Nitrogen-Carbon Electrocatalyst Provides a Reversible Electron Transfer Platform for Efficient Uranium Extraction from Seawater. Adv. Mater. 2021, 33 (51), e2106621 10.1002/adma.202106621. 34599784

[ref5] Wang C.; Helal A. S.; Wang Z.; Zhou J.; Yao X.; Shi Z.; Ren Y.; Lee J.; Chang J. K.; Fugetsu B.; Li J. Uranium In Situ Electrolytic Deposition with a Reusable Functional Graphene-Foam Electrode. Adv. Mater. 2021, 33 (38), e2102633 10.1002/adma.202102633. 34346102

[ref6] Abney C. W.; Mayes R. T.; Saito T.; Dai S. Materials for the Recovery of Uranium from Seawater. Chem. Rev. 2017, 117 (23), 13935–14013. 10.1021/acs.chemrev.7b00355. 29165997

[ref7] Xie Y.; Liu Z.; Geng Y.; Li H.; Wang N.; Song Y.; Wang X.; Chen J.; Wang J.; Ma S.; Ye G. Uranium Extraction from Seawater: Material Design, Emerging Technologies and Marine Engineering. Chem. Soc. Rev. 2023, 52 (1), 97–162. 10.1039/D2CS00595F. 36448270

[ref8] Liu Z.; Lan Y.; Jia J.; Geng Y.; Dai X.; Yan L.; Hu T.; Chen J.; Matyjaszewski K.; Ye G. Multi-scale Computer-aided Design and Photo-Controlled Macromolecular Synthesis Boosting Uranium Harvesting from Seawater. Nat. Commun. 2022, 13 (1), 3918–3930. 10.1038/s41467-022-31360-x. 35798729 PMC9262957

[ref9] Keener M.; Hunt C.; Carroll T. G.; Kampel V.; Dobrovetsky R.; Hayton T. W.; Menard G. Redox-switchable Carboranes for Uranium Capture and Release. Nature. 2020, 577 (7792), 652–655. 10.1038/s41586-019-1926-4. 31969700

[ref10] Zhang S.; Chen L.; Qu Z.; Zhai F.; Yin X.; Zhang D.; Shen Y.; Li H.; Liu W.; Mei S.; Ji G.; Zhang C.; Dai X.; Chai Z.; Wang S. Confining Ti-oxo Clusters in Covalent Organic Framework Micropores for Photocatalytic Reduction of the Dominant Uranium Species in Seawater. Chem 2023, 9, 3172 10.1016/j.chempr.2023.06.008.

